# [Corrigendum] Molecular impact of bone morphogenetic protein 7, on lung cancer cells and its clinical significance

**DOI:** 10.3892/ijmm.2025.5585

**Published:** 2025-07-14

**Authors:** Yinan Liu, Jinfeng Chen, Yue Yang, Lijian Zhang, Wen G. Jiang

Int J Mol Med 29: 1016-1024, 2012; DOI: 10.3892/ijmm.2012.948

Following the publication of the above article, an interested reader drew to the authors' attention that, for the electrophoretic blots shown in Fig. 3A-a, the data shown for the BMP7 and GAPDH bands were strikingly similar, such that it appeared that the same data had been included in this figure part to show the results from the differently performed experiments.

The authors were able to re-examine their original data, and realized that the BMP7 bands had inadvertently been included in this figure twice. The revised version of Fig. 3, now incorporating the correct data for the GAPDH bands in Fig. 3A-a, is shown on the next page. The authors can confirm that the error made in asembling this figure did not have a significant impact on either the results or the conclusions reported in this study, and all the authors agree with the publication of this Corrigendum. The authors are grateful to the Editor of *International Journal of Molecular Medicine* for allowing them the opportunity to publish this Corrigendum; furthermore, they apologize to the readership of the Journal for any inconvenience caused.

## Figures and Tables

**Figure 3:**
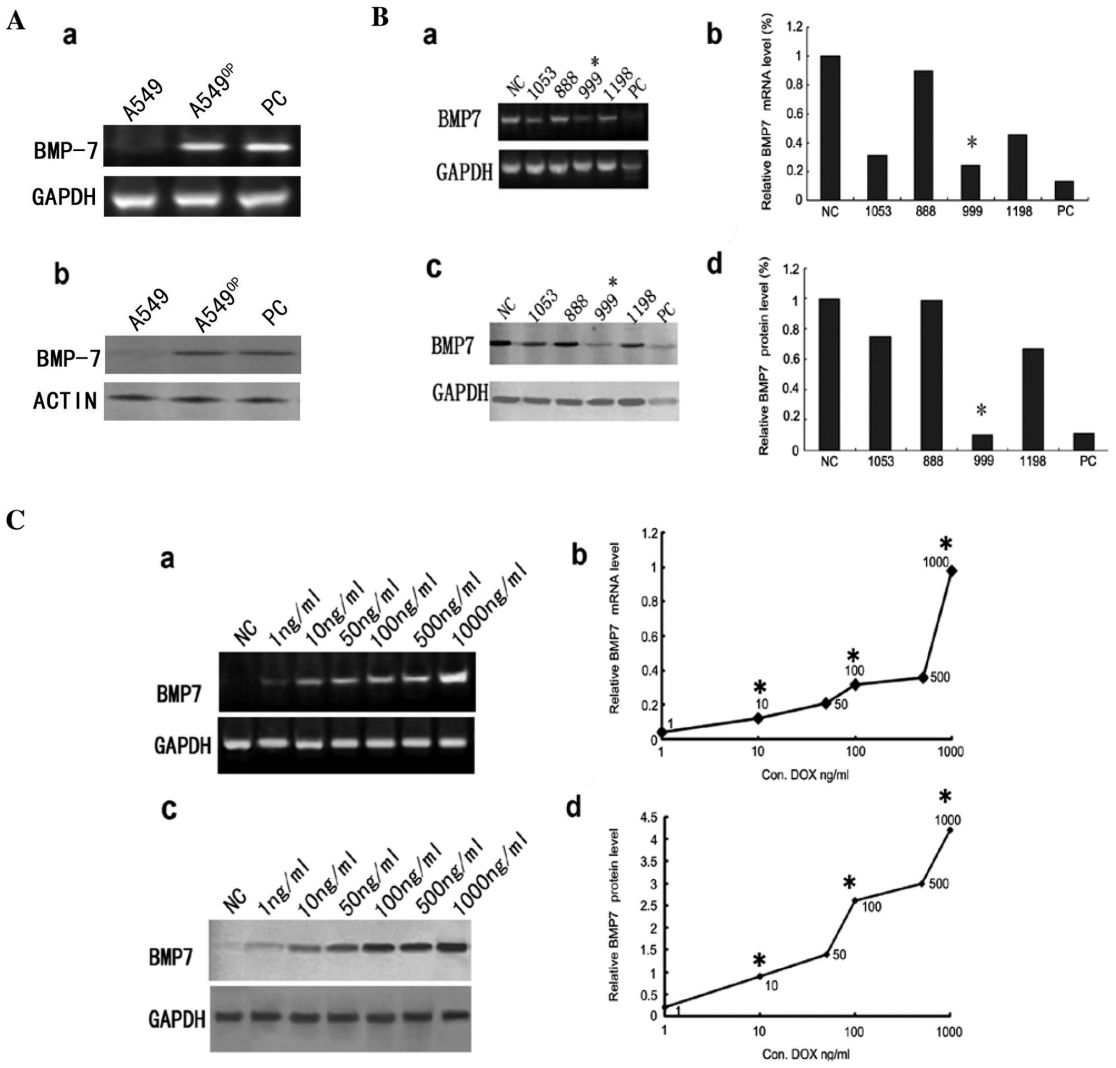
Reduced and induced expression and overexpression of BMP7 protein in lung cancer cells. (A) A549 cells transfected with BMP7 plasmid (A549OP cells) expressed BMP7 as shown using the RT-PCR method (Aa and b) and western blot analysis (PC, positive control). Expression status of BMP7 transcripts in SPC-A1 cells through shRNA-mediated depletion of BMP7 mRNA was strongly decreased for shRNA target 999 when compared with the other targets (1053, 888, 1198). (Ba and c) shRNA designed for four different targets could reduce BMP7 expression in SPC-A1 cell using RT-PCR and western blot analysis. (Bb and d) Normalised band volumes of BMP7 expression in SPC-A1 cells. (C) Induced expression levels of BMP7 in pulmonary tumour cell lines by RT-PCR and western blot analysis using GAPDH expression as the control. Three DOX concentrations were chosen for the induction model (10, 100 and 1,000 ng/ml).

